# Rapid and specific detection of wheat spindle streak mosaic virus using RT-LAMP in durum wheat crude leaf extract

**DOI:** 10.1371/journal.pone.0299078

**Published:** 2024-02-29

**Authors:** Monica Marra, Paolo Mussano, Eugenio Pinton, Cinzia Montemurro, Elena Baldoni, Claudio Ratti, Slavica Matić, Chiara D’Errico, Gian Paolo Accotto

**Affiliations:** 1 Institute for Sustainable Plant Protection, National Research Council, Turin, Italy; 2 Department of Soil, Plant and Food Science, University of Bari Aldo Moro, Bari, Italy; 3 Department of Agriculture, Forestry and Food Science, University of Turin, Turin, Italy; 4 Institute for Sustainable Plant Protection, National Research Council, Bari, Italy; 5 Institute of Agricultural Biology and Biotechnology, National Research Council, Milan, Italy; 6 Department of Agricultural and Food Sciences, University of Bologna, Bologna, Italy; Pan African University of Life and Earth Sciences Institute, PAULESI, NIGERIA

## Abstract

To accurately determine the spread of any pathogen, including plant viruses, a quick, sensitive, cost-effective, point-of-care diagnostic assay is necessary. Wheat spindle streak mosaic virus (WSSMV) is a *Bymovirus*, transmitted by the plasmodiophorid *Polymyxa graminis* Led, which causes yellow mosaic and reduces the grain yield in wheat. Currently, detection protocols for WSSMV use ELISA or more sensitive PCR-based approaches requiring specialized laboratory and personnel. A protocol for reverse transcription loop mediated isothermal amplification (RT-LAMP) has been developed and optimized for the rapid detection of viruses using crude extracts from wheat leaves. The protocol was specific for WSSMV detection, while no reaction was observed with SBCMV or SBWMV, the non-target viruses transmitted by the same vector. The RT-LAMP assay was shown to be as sensitive as the one-step WSSMV specific RT-PCR. The RT-LAMP assay can be performed under field conditions using a portable instrument, and can help the actual spread of WSSMV, an aspect of this virus not yet well understood, to be explored.

## Introduction

Durum wheat (*Triticum turgidum* L. subsp. *durum* (Desf.) Husn.) is an important cereal crop, accounting for almost 15% of Europe’s total wheat production. In countries where production is more concentrated, it is one of the most important sources of carbohydrates [1]. Durum wheat represents the most prevalent staple crop in the Mediterranean basin, with Italy as the largest European producer [2–4].

In common with other cereal crops, the productivity of durum wheat is affected by various biotic and abiotic stresses. The incidence of viral pathogens on durum wheat and other wheat species is generally not high, but several studies have recently reported an increased prevalence of wheat viruses in Europe [5, 6], especially soil-borne viruses [7, 8], *e*.*g*. wheat spindle streak mosaic virus (WSSMV). It is one of the most important wheat viruses, belonging to the genus *Bymovirus* in the family *Potyviridae* [9], and causes significant yield losses and reductions in grain quality [10, 11]. WSSMV also infects other cereals such as common wheat (*Triticum aestivum*), triticale (× Triticosecale) and rye (*Secale cereale*), while does not attack dicotyledonous plant species [12–14].

The plasmodiophorid *Polymyxa graminis* Led. is the only vector of WSSMV, and transmission occurs mainly in autumn and spring, 15°C being the optimum temperature [15, 16]. Transmission by *P*. *graminis* ensures WSSMV persistence in the soil inside the vector’s resting spores, which complicates disease management [12]. *P*. *graminis is the vector also of* two other viruses: soil-borne cereal mosaic virus (SBCMV) and soil-borne wheat mosaic virus (SBWMV), the most detrimental wheat viruses, both belonging to the genus *Furovirus* [8]. The infections by these three viruses can be simultaneously present in the same field. WSSMV virus was first detected in Canada [17] and is widely distributed across most continents, including North America [18], Europe [7, 12, 14, 19–21], Africa [22], and Asia [23]. Although reported in Italy [19], data about its current spread are still very limited.

As there are no direct chemical or agronomic methods available to contain WSSMV-induced disease, preventive measures must be taken. These include selecting vector-free soils and growing resistant cultivars [8]. Timely detection of the virus is vital to improve disease management. This requires the use of fast, sensitive, and specific molecular techniques to detect WSSMV, in both symptomatic and asymptomatic plant materials. Current molecular diagnostics for WSSMV consists of conventional techniques, such as RT-PCR and real-time PCR [12, 24, 25], which are limited by low test velocity, restricting the possibility of in-field analyses.

The loop-mediated isothermal amplification (LAMP) technique amplifies DNA under constant temperature in short times, without requiring a thermal cycler [26]. LAMP offers high sensitivity, enabling the detection of pathogens at low concentrations [27–29]. Moreover, thanks to the high resilience of the polymerase used in LAMP, crude plant extracts obtained by simple fragmentation and homogenization of plant tissues in a specific buffer can be used, without requiring nucleic acid extraction [27, 29].

LAMP is used for the detection of many plant pathogens [26], and in the form of reverse transcription LAMP (RT-LAMP) for RNA viruses. Our research groups have recently developed specific LAMP and RT-LAMP protocols for SBCMV detection [27]. However, to date no isothermal amplification assay has been available to detect WSSMV. To contribute to the better management of WSSMV-induced disease through timely, specific, quick, and sustainable virus detection, a WSSMV-specific RT-LAMP procedure has been developed. The goal was to produce a RT-LAMP assay applicable to crude plant extracts which could be concluded in minutes using a portable battery-powered instrument, thus allowing in-field application. The developed assay will contribute to large-scale analyses of WSSMV spread, rapid evaluation of new viral outbreaks in wheat fields, and the application of timely management strategies.

## Materials and methods

### Plant materials

Leaf samples of five durum wheat plants were collected in an experimental station at the University of Bologna (Cadriano Experimental Center), Italy, and used as positive controls, following a preliminary end-point RT-PCR according to previous work [10]. The WSSMV isolate CAD22-W1, used to develop the assay described in this paper, was obtained from plants collected in the same field. The negative control consisted of a pool of healthy leaves obtained from durum wheat plants derived from seeds collected in fields not infested by *P*. *graminis*. For the development of the RT-LAMP protocol, a total of six samples were used. These included one positive control and one negative control for optimizing the reaction temperature, as well as all five positive controls for dilution assays.

Following the results obtained for the positive controls, leaf samples were collected from 26 plants grown in a screenhouse for four months in pots containing soil derived from the above-mentioned infested field.

### RNA extraction and crude extract preparation

The Spectrum™ Plant Total RNA Kit (Sigma-Aldrich, St. Louis, MO, USA) was used for total RNA extraction from durum wheat leaf samples (100 mg). RNA was eluted in nuclease-free water (50 μL) and the concentration determined using a NanoDrop™ 2000 Spectrophotometer (ThermoFisher, Waltham, MA, USA).

The crude extracts were prepared using TET buffer [30], previously found to be optimal for the LAMP analysis [27]. Durum wheat leaves (100 mg) were mincedin TET buffer (1 mL) using tubes with one extraction bead (Plant material DNA extraction kit, OptiGene, West Sussex UK), then crude extracts were diluted in sterile water [27].

### RT-LAMP assay

Primer Explorer V5 (Eiken Chemical Co. Ltd., Tokyo, Japan) software was used to design LAMP primers, based on the RNA-1 sequence of WSSMV isolate CAD22-W1 (GenBank OQ945144) encoding the nuclear inclusion protein b (NIb) (Table 1).

**Table 1:** RT-LAMP primers for detection of WSSMV.

Primer set	Name	Sequence (5’-3’)	Position (nt)	Concentration used in LAMP reaction
**NIb**	F3_NIb	AATGCAATGGGCGAGAAG	6205–6222	0.25 μM
	B3_NIb	ATGCAAACGGTAAACATCAA	6399–6418	0.25 μM
	FIP_NIb	GCTTTGGCGTGTTAAAAGACTCAT-GGTGTTCTTCATTCGTATTTAGC	6267–6290 (F1c_NIb) + 6227–6249 (F2_NIb)	2.50 μM
	BIP_NIb	TTGACTGAAGAATACGAAGCCG-ATGGAAGGGATTGGGAGA	6323–6344 (B1c_NIb) + 6379–6396 (B2_NIb)	2.50 μM
	LOOPB_NIb	ATATCCTTGCTGCCATGAAGGA	6345–6366	1.25 μM

The position refers to the GenBank sequence OQ945144. FIP_NIb (BIP_NIb)primer is build by F1c_NIb and F2_NIb (B1c_NIb and B2_NIb) ones. All five primers were used for the RT-LAMP protocol.

The instruments Bio-Rad CFX96 Real-Time PCR Detection System (Bio-Rad, Hercules, CA, USA), or Hyris bCUBE (Hyris, London, UK), have been used to run the analysis. Previous work [27] showed that LAMP protocols yield comparable results when performed on either instrument. Two microlitres of crude extract were used as a template. The Isothermal Master Mix ISO-004® (OptiGene, Horsham, UK) was used to perform the reaction, in a final volume of 10 μL. The primer concentrations is indicated in Table 1. To improve the reaction’s performance, 0.5 U of Avian Myeloblastosis Virus Reverse Transcriptase (AMV RT) was added to the mix. For each biological replicate three technical replicates were performed.

To determine the best reaction temperature, RT-LAMP assays were performed on crude extract between 60 and 65°C (increasing by 1°C for each test). The RT-LAMP reaction lasted 40 minutes, while melting curves were run from 65 to 95°C (at increments of 0.5°C).

### One-step RT-PCR on crude extract

One-step RT-PCR reactions were run with the One-Step RT-PCR Kit (Qiagen, Hilden, Germany) using the external primers F3_NIb and B3_NIb designed for the RT-LAMP protocol (Table 1). The reaction mix included 0.6 μL of each primer (10 μM), 4 μL of nuclease-free water, 2 μL of 5x QIAGEN One-Step RT-PCR Buffer, 0.4 μL of 2X dNTP Mix (containing 10 mM of each dNTP), 0.4 μL of QIAGEN One-Step RT-PCR Enzyme Mix and 2 μL of crude extract as template, in a final volume of 10 μL. Thermal cycling conditions were 50°C for 30 min, 95°C for 15 min, followed by 36 cycles of 94°C for 30 sec, 63°C for 30 sec, and 72°C for 1 min, concluding with 70°C for 10 min. The amplified product was run in a 1% agarose gel containing RedSafe Nucleic Acid Staining Solution (iNtRON Biotechnology, Seongnam, Republic of Korea) and observed with the Gel Documentation System (UVITEC, Cambridge, UK).

### Sequence analyses: Multiple alignments and similarity search

WSSMV has been reported in several countries; making it important to verify whether the new diagnostic protocol detects all the isolates described. The RNA1 sequence of the isolate CAD22-W1 (GenBank OQ945144), used for primer design, was thus aligned with the other WSSMV sequences present in GenBank (OP357940, X73883, MN046368, MN046367, MH645041, MH645040) in the genomic region where the LAMP primers were designed. To achieve multiple alignment, the Clustal Omega program on the EBI platform (https://www.ebi.ac.uk/Tools/msa/clustalo/) was used. Further, the region encompassing Primer set NIb was used to search for high-similarity sequences in the GenBank, using BLASTN with the default algorithm parameters.

### Protocol specificity

Analytical specificity, i.e. the ability of the RT-LAMP to detect the target viral sequences rather than others, was tested on total RNA obtained from plants infected by the target organism WSSMV (CAD22-W1 Italian isolate and DSMZ PV-0541 German isolate; GenBank OP357940), and from plants infected by the non-target organisms SBCMV (PLAVIT-CAD22 isolate from Italy) and SBWMV (provided by Dr. A. Niehl, Julius Kühn-Institut, Germany). Each assay was run in triplicate (technical replicates).

### Optimization of crude extract dilutions for one-step RT-PCR and RT-LAMP

The introduction of novel diagnostic assays requires comparison with commonly used methods, particularly in terms of sensitivity, cost, and rapidity. The sensitivity of RT-LAMP was compared with that of one-step RT-PCR, the most commonly used technique.

To evaluate the best dilution to run the analysis, and the limit of detection (LOD) of the assay, crude extracts prepared from five positive plant samples were serially diluted (10^−3^ to 10^−7^) and tested using RT-LAMP and one-step RT-PCR. After selecting the optimal dilutions, 26 samples from plants grown in infected soil in a screenhouse were tested. Crude extract prepared from leaves of uninfected healthy durum wheat plants was used as negative control, while crude extract isolated from WSSMV-infected wheat leaves was used as positive control. A no-template control (NTC), with sterile water as a template, was also included.

## Results

### Optimization of RT-LAMP reaction conditions

**Fig 1:**
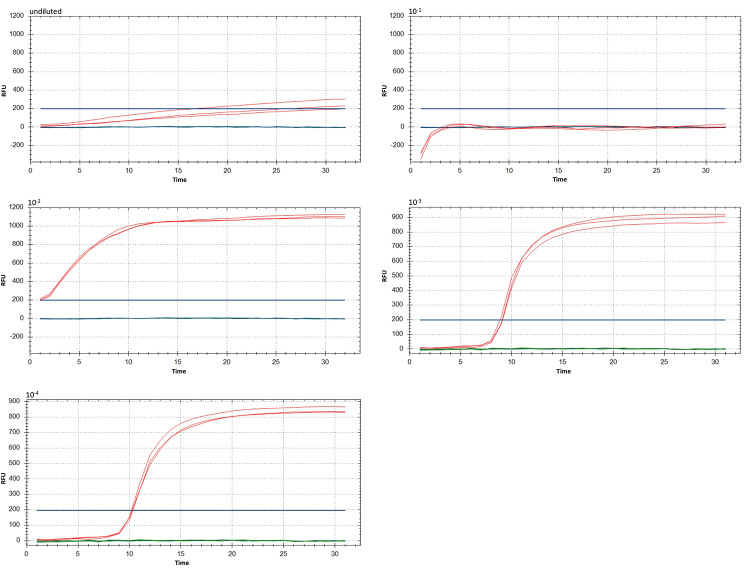
RT-LAMP reaction on dilutions of crude extract (from undiluted to 10^−4^ dilution). The RT-LAMP reaction was performed at 63°C for 40 minutes in the CFX instrument. Red = WSSMV positive control; blue = negative control (healthy plant); green = NTC (no-template control—water); Three technical replicates were run for each sample. RFU = Relative fluorescence units.

To determine the best reaction conditions, the RT-LAMP protocol was tested on crude extracts obtained from positive (WSSMV-infected) and negative (healthy) control plants. A preliminary test at 63°C was run using different dilutions of crude extract (Fig 1). Dilutions 10^−3^ and 10^−4^ yielded optimal results, with good reproducibility of technical replicates, short reaction time (Rt), and no reaction with negative or with no-template controls. The 10^−4^ dilution was used to create a temperature gradient.

**Fig 2:**
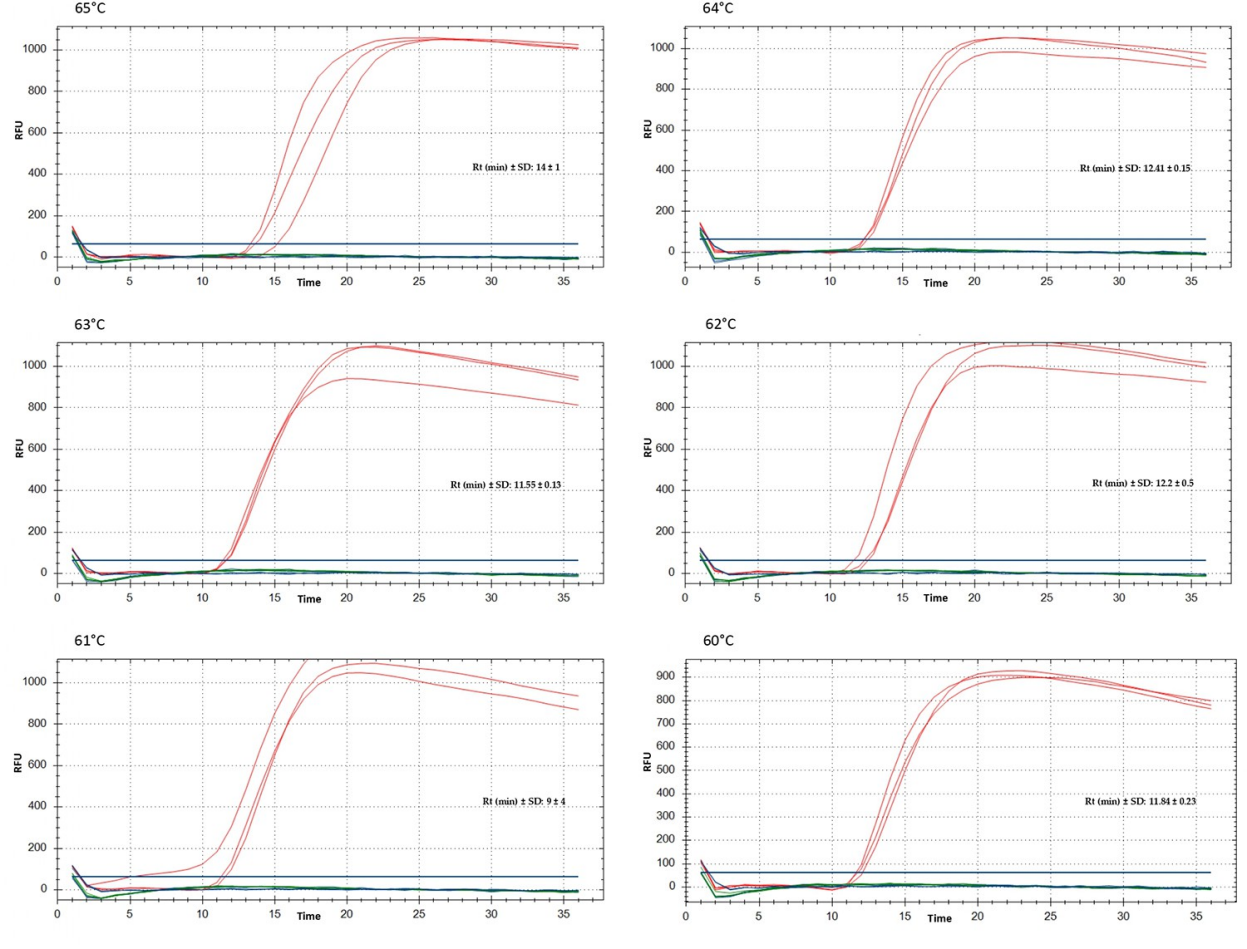
RT-LAMP temperature gradient tests for the detection of WSSMV. The experiment was run from 60 to 65°C on crude extract at 10^−4^ dilution in the CFX instrument. Three technical replicates were done for each sample. Red = WSSMV positive control; blue = negative control (healthy plant); green = no-template (water); RFU = Relative fluorescence units; Rt: reaction time; SD: standard deviation.

The temperature gradient assay from 60 to 65°C produced positive signals at all temperatures with the positive control (WSSMV-infected plant), with Rt below 15 minutes (Fig 2), while negative control (healthy plant) yielded no amplification. The temperature of 63°C was chosen because it combined short Rt with good reproducibility of the three technical replicates. The running time of the RT-LAMP assay used for further experiments was set to 30 minutes, to allow detection of WSSMV in samples with virus concentrations below those in the positive control.

### Choice of the best dilution and sensitivity comparison between RT-LAMP and one-step RT-PCR

**Fig 3:**
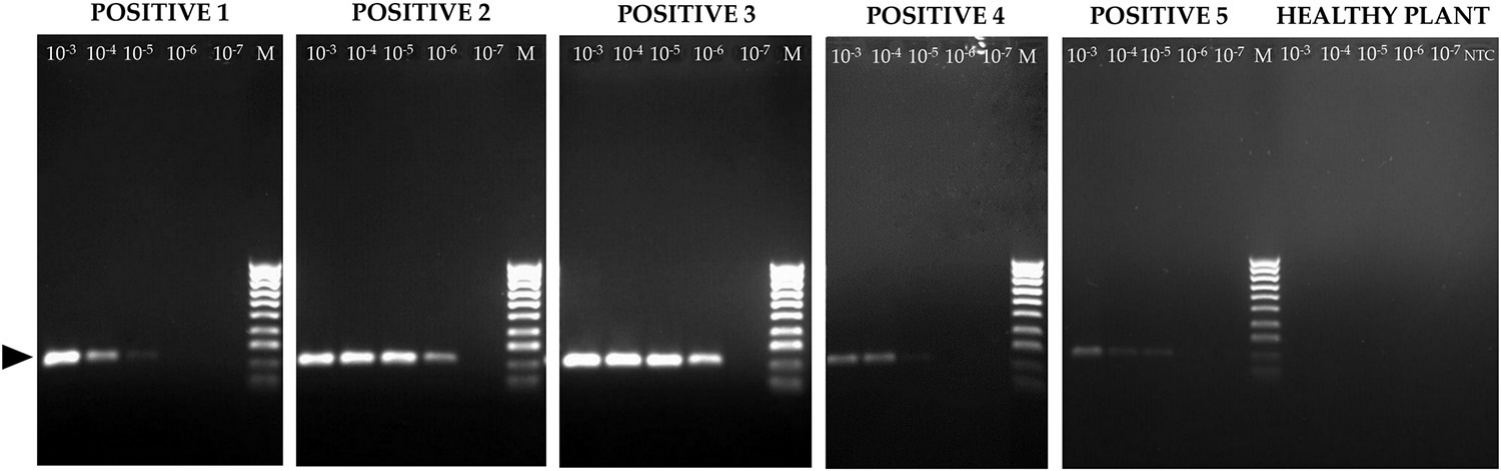
One-step RT-PCR on serial dilutions of crude extracts from five WSSMV-infected wheat plants. The black arrow identifies the amplified fragment (213 bp). Healthy plant: negative control; M: HyperLadder™ 100bp (Meridian Bioscience, U.S.A.) containing 10 regularly spaced bands, ranging from 100 bp to 1013 bp; NTC: no template control (water).

Crude extracts prepared from five WSSMV-infected durum wheat plants (positive samples) and from one uninfected negative control were diluted from 10^−3^ to 10^−7^ in sterile water and analysed with both one-step RT-PCR and RT-LAMP techniques (Table 2, Fig 3).

**Table 2:** RT-LAMP and one-step RT-PCR of crude extracts from five WSSMV-infected wheat plants.

Sample	Crude extract dilution	RT-LAMP on crude extract	One-step RT-PCR on crude extract
		Rt (min) ± SD	+/-
**POSITIVE 1**	10^−3^	9.5 ± 0.4	+
	10^−4^	10.42 ± 0.20	+
	10^−5^	11.1 ± 0.8	+
	10^−6^	nd	-
	10^−7^	nd	-
**POSITIVE 2**	10^−3^	9.94 ± 0.10	+
	10^−4^	10.8 ± 1.2	+
	10^−5^	12.4 ± 0.4	+
	10^−6^	13.9 ± 0.4	+
	10^−7^	nd	-
**POSITIVE 3**	10^−3^	11.7 ± 0.4	+
	10^−4^	15.0 ± 1.2	+
	10^−5^	14.8 ± 2.4	+
	10^−6^	nd	+
	10^−7^	nd	-
**POSITIVE 4**	10^−3^	10.99 ± 0.28	+
	10^−4^	13.1 ± 1.8	+
	10^−5^	14.50 ± 2.20	+
	10^−6^	15 ± 3	-
	10^−7^	nd	-
**POSITIVE 5**	10^−3^	10.8 ± 0.4	+
	10^−4^	11.8 ± 0.7	+
	10^−5^	14.4 ± 2.0	+
	10^−6^	nd	-
	10^−7^	nd	-
**NEGATIVE**	10^−3^	nd	-
	10^−4^	nd	-
	10^−5^	nd	-
	10^−6^	nd	-
	10^−7^	nd	-

RT-LAMP was performed in bCUBE. The melting temperature of RT-LAMP was 84.9 ± 0.3°C. Three technical replicates were carried out for each sample. Negative: healthy plant; Rt: reaction time; SD: standard deviation; nd: not detected; +: positive reaction;—: negative reaction.

The one-step RT-PCR yielded the expected DNA amplicon of 213 bp (Fig 3) from the five positive samples. With this assay, three samples tested positive up to the 10^−5^ dilution and two up to the 10^−6^ dilution. The RT-LAMP performed on the same crude extracts detected WSSMV in three samples up to the 10^−5^ dilution and in two up to 10^−6^ dilution, with reaction times ranging between 9.5 and 15.3 minutes (Table 2). Comparison of serial dilutions of crude extracts between two assays showed the two assays to have comparable sensitivity.

For subsequent experiments the 10^−3^ dilution of crude extract was used, rather than extreme dilutions, because virus concentration in plants vary widely and extreme dilutions may lead to false-negative results. Further, at the 10^−3^ dilution, the virus was detected more quickly (9.5 vs. 11.7 min) (Table 2).

### Specificity tests and multiple alignment

**Fig 4:**
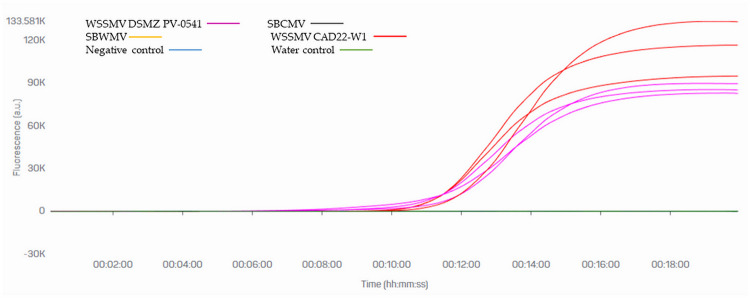
RT-LAMP specificity test. The test was run on RNA from samples infected with WSSMV CAD22-W1 (positive control), WSSMV DSMZ PV-0541, SBCMV and SBWMV, all diluted 10^−3^. Negative control: healthy plant; Water control: No-template control; the bCUBE instrument was used, with three technical replicates. Some lines are not visible in the graph because the fluorescence they produced did not exceed that of the no-template control.

The primer set design being based on the CAD22-W1 virus isolate, it was evaluated whether the optimized RT-LAMP assay could efficiently detect different WSSMV isolates. Reactions were run on RNA extracted from the WSSMV isolates CAD22-W1 and DSMZ PV-0541 used previously. Amplification signals were observed in both WSSMV isolates PV-0541 and CAD22-W1, despite the mismatches in the portions covered by primers (Fig 4).

**Fig 5:**
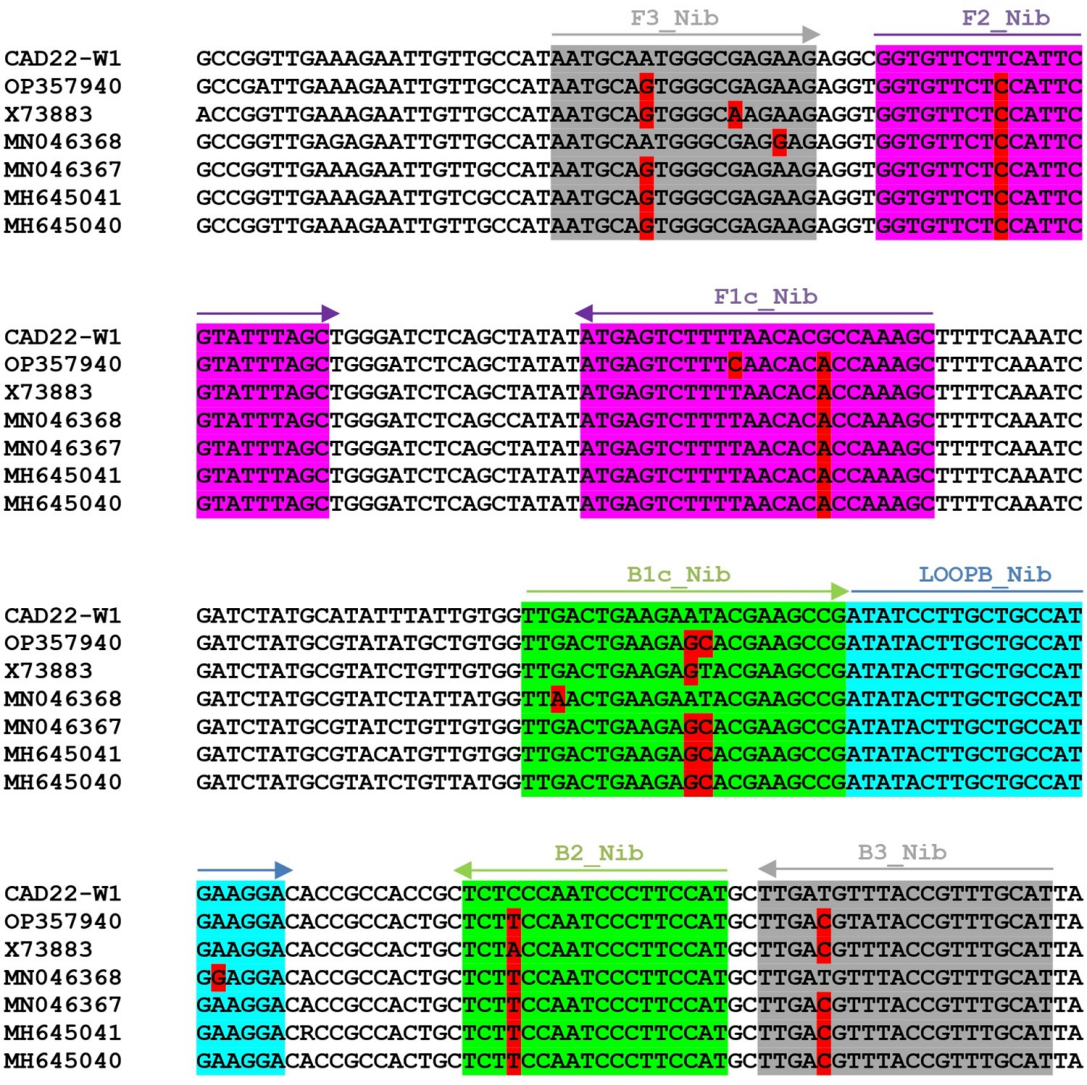
Sequence alignment of a portion of WSSMV RNA 1 from seven WSSMV isolates available in GenBank. Nucleotide differences from CAD22-W1 (in the portions covered by primers) are highlighted in red. Arrows indicate the position of the RT-LAMP primers.

When a multiple sequence alignment was run with all WSSMV RNA 1 sequences available in GenBank (Fig 5), the five WSSMV isolates not experimentally tested appeared close to DSMZ PV-0541, meaning that it is probable those isolates can also be detected.

Under field conditions, WSSMV is often found in association with other soil-borne viruses of cereals, such as SBCMV and SBWMV. Reactions run on RNA of SBCMV and SBWMV produced no amplification, thus ruling out the possibility of the RT-LAMP assay recognizing these viruses (Fig 4).

### Similarity search: *In silico* BLASTN analysis

Specificity is an important feature of a diagnostic protocol, thus an *in silico* BLASTN analysis of the region encompassing the RT-LAMP primers was done to search for highly similar sequences. Sequence identity with the other WSSMV accessions was 92.5% - 94%, but only around 75% with barley yellow mosaic virus (BYMV) and wheat yellow mosaic virus (WYMV), other members of the *Bymovirus* genus. Although these viruses could not be tested in the laboratory, the marked decrease in sequence similarity makes it very unlikely that they would be detected by the WSSMV-specific test developed here. For example, within the region of the RT-LAMP primers, 32 mismatches were detected in BYMV reference sequence NC_002990 (S1 Fig).

### Application of RT-LAMP on unknown samples

**Fig 6:**
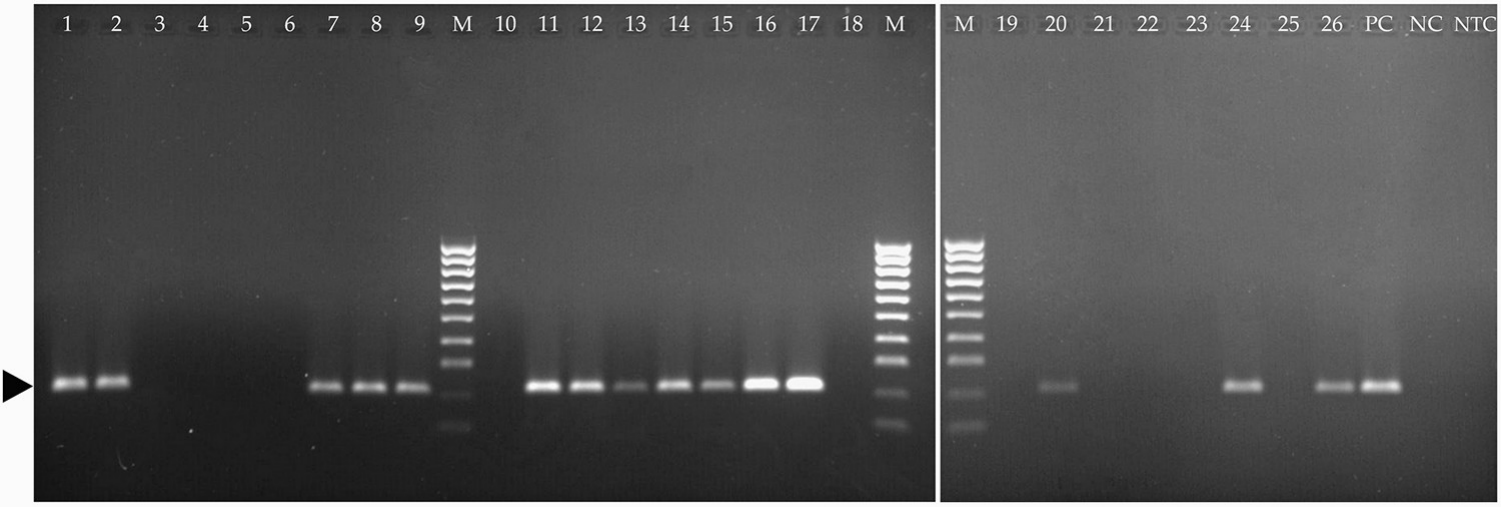
One-step RT-PCR on crude extracts of leaf samples from durum wheat plants grown in virus-infected soil. The black arrow identifies the amplified fragment (213 bp). M: HyperLadder™ 100bp (Meridian Bioscience, U.S.A.) containing 10 regularly spaced bands, ranging from 100 bp to 1013 bp; PC: Positive control; NC: Negative Control (healthy plant); NTC: No template control (water).

Having demonstrated the validity of the RT-LAMP on the five positive samples reported above, the leaves of wheat plants suspected of being naturally infected by WSSMV were analysed. One-step RT-PCR and RT-LAMP assays were used to test the crude extracts from 26 samples of wheat plants grown in infected soil (Table 3, Fig 6). Since the virus concentration in field samples can vary greatly, the 10^−3^ dilution of crude extract was used for the analyses, rather than higher dilutions.

**Table 3:** Comparison between one-step RT-PCR and RT-LAMP.

	RT-LAMP on crude extract	One-step RT-PCR on crude extract
Sample	Rt (min) ± SD	+/-
**1**	9.5 ± 0.4	+
**2**	9.94 ± 0.10	+
**3**	nd	-
**4**	nd	-
**5**	nd	-
**6**	nd	-
**7**	13.11 ± 0.20	+
**8**	11.7 ± 0.4	+
**9**	12.9 ± 0.3	+
**10**	nd	-
**11**	10.7 ± 0.8	+
**12**	14.8 ± 1.9	+
**13**	13.12 ± 0.18	+
**14**	12.53 ± 0.17	+
**15**	13.0 ± 0.3	+
**16**	11.8 ± 0.3	+
**17**	11.78 ± 0.20	+
**18**	nd	-
**19**	nd	-
**20**	12.5 ± 0.7	+
**21**	nd	-
**22**	nd	-
**23**	nd	-
**24**	12.6 ± 0.7	+
**25**	nd	-
**26**	13.70 ± 0.20	+
**Positive**	11.4 ± 0.6	+
**Negative**	nd	-
**NTC**	nd	-

Reactions performed on crude extracts (10^−3^ dilution) of leaf samples from durum wheat plants grown in virus-infected soil. Three technical replicates were carried out for each sample.

Negative: healthy plant; NTC: no-template control (water); Rt: reaction time; SD: standard deviation; nd: not detected; +: positive reaction;—: negative reaction.

Analysis of crude extracts found 15 out of 26 plants positive for WSSMV with either one-step RT-PCR or RT-LAMP (Table 3 and Fig 6). The RT-LAMP technique generated a positive signal with Rt between 9.5 and 14.8 min, while the one-step RT-PCR produced a 213 bp amplicon. These results, using similar amounts of starting material, showed that the two tests produced identical results: 15 infected and 11 non-infected plants. The RT-LAMP assay developed here thus displays the same detection efficiency using crude extracts as one-step RT-PCR.

## Discussion

Among innovative methods developed to detect plant pathogens, the LAMP assay is now widely and increasingly used [31]. This technique has demonstrated excellent sensitivity, rapidity, and specificity; a further advantage is that it does not require the extraction of nucleic acids, enabling it to be used directly in the field. RT-LAMP assays have been described for several plant viruses with RNA genomes [26], including wheat viruses [32], but not for WSSMV. LAMP-based protocols for wheat virus detection described to date mainly entail the extraction of DNA [33, 34] or RNA [35] or reverse transcription into cDNA [36], few of them being directly applicable to crude plant extracts [27, 37]. This study developed a protocol of RT-LAMP for the direct detection of WSSMV in 30 minutes from crude durum wheat leaf extracts. The NIb primer set performed well even on crude extracts, without reacting with the control sample from healthy plants. Other soil-borne viruses, such as SBCMV and SBWMV, are frequently found together with WSSMV in fields [19], meaning it was important to show there was no cross-reaction with these viruses. The assay did not detect any of these viruses, thus it was specific for WSSMV (Fig 4).

Another important point was to determine whether the proposed system can detect WSSMV isolates other than CAD22-W1. The assay was run on the isolate DSMZ PV-0541, whose RNA-1 has an overall sequence identity of 93% with CAD22-W1 (Fig 5); the result showed that this non-identical isolate was efficiently recognized (Fig 4). It was not possible to test other isolates. However, a multiple alignment based on the genomic region where primers were located showed PV-0541 to possess high similarity with other WSSMV isolates (Fig 5), suggesting that these other isolates may also be readily detected by the LAMP assay.

The RT-PCR and the RT-LAMP assay both showed similar LODs and sensitivity levels (Table 2, Fig 3); 10^−3^ dilution was optimal for detection of WSSMV with both techniques. The detection efficiency of the RT-LAMP assay was found to be comparable to that of one-step RT-PCR on the crude extracts. When 26 samples were tested to investigate whether they were infected by WSSMV (Table 3, Fig 6), the results (15 positives and 11 negatives) for the two assays were closely comparable. This showed that the RT-LAMP protocol on crude extracts, as designed and optimized here, can replace slower and laborious techniques such as one-step RT-PCR for the detection of WSSMV.

## Conclusions

The proposed RT-LAMP protocol has all the features appropriate for point-of-care testing: i) it is run on crude extracts obtained with no toxic or harmful chemical buffers; ii) it is run on a portable instrument; iii) results are obtained in minutes; iv) cost of consumables is estimated to be less than one euro per sample; v) the detection efficiency is comparable to that of one-step RT-PCR on crude extracts; vi) the test specifically recognizes WSSMV.

In conclusion, the assay is ready for extensive surveys aimed at determining the actual pathogenicity and spread of WSSMV. Awareness of its presence can help farmers to use alternative crops or wheat-resistant genotypes as valid disease management options. Further, this fast detection protocol can be applied to study the susceptibility/resistance of different wheat genotypes.

## Supporting information

S1 Fig *In silico* BLASTN analysis. Alignment between WSSMV-CAD-W1 (query) and barley yellow mosaic virus reference sequence NC_002990 (subject) in the region encompassing LAMP Primer set NIb.
(DOCX)

S2 Fig Original, uncropped electrophoresis gel picture underlying Fig 3 from the main text. (DOCX)

S3 Fig Original, uncropped electrophoresis gel picture underlying Fig 6 from the main text. (DOCX)
