# Supplementary material for: Rapid and specific detection of wheat spindle streak mosaic virus using RT-LAMP in durum wheat crude leaf extract
Source: PLoS One. 2024 Feb 29;19(2):e0299078. doi: 10.1371/journal.pone.0299078 (PMC10903832; doi:10.1371/journal.pone.0299078)

**S1 Fig. *In silico* BLASTN analysis**.

Alignment between WSSMV-CAD-W1 (query) and Barley yellow mosaic virus reference sequence NC_002990 (subject) in the region encompassing LAMP primer set NIb. Primer sequences are boxed.


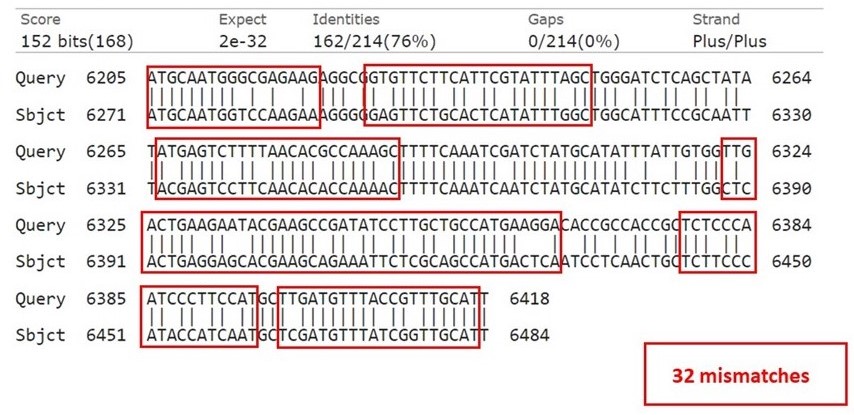

Supplement: S1 Fig — Alignment between WSSMV-CAD-W1 (query) and barley yellow mosaic virus reference sequence NC_002990 (subject) in the region encompassing LAMP Primer set NIb. (DOCX) [file pone.0299078.s001.docx]
