# Supplementary material for: Rapid and specific detection of wheat spindle streak mosaic virus using RT-LAMP in durum wheat crude leaf extract
Source: PLoS One. 2024 Feb 29;19(2):e0299078. doi: 10.1371/journal.pone.0299078 (PMC10903832; doi:10.1371/journal.pone.0299078)
Supplement: S2 Fig — (DOCX) [file pone.0299078.s002.docx]

**S2 Fig. Original, uncropped electrophoresis gel picture underlying Fig 3 from the main text.**

1. POSITIVE 1, POSITIVE 2, POSITIVE 3
2. POSITIVE 4, POSITIVE 5, HEALTHY PLANT, NTC

**A**





**B**
