# Supplementary material for: Rapid and specific detection of wheat spindle streak mosaic virus using RT-LAMP in durum wheat crude leaf extract
Source: PLoS One. 2024 Feb 29;19(2):e0299078. doi: 10.1371/journal.pone.0299078 (PMC10903832; doi:10.1371/journal.pone.0299078)
Supplement: S3 Fig — (DOCX) [file pone.0299078.s003.docx]

**S3 Fig. Original, uncropped electrophoresis gel picture underlying Fig 6 from the main text.**

1. Samples 1-18
2. Samples 19-26, Positive control, Negative control and No Template Control

**A**





**B**
